# The Parasitoid Complex of *Aleurothrixus floccosus* (Hemiptera: Aleyrodidae) in the Citrus Groves of Central–Southern Italy

**DOI:** 10.3390/insects16101037

**Published:** 2025-10-09

**Authors:** Gianluca Melone, Lucia Andretta, Valentino Maria Guastaferro, Eleonora Romito, Giorgio Formisano, Massimo Giorgini, Stefania Laudonia

**Affiliations:** 1 Institute for Sustainable Plant Protection (IPSP)—CNR, P.le Enrico Fermi 1, 80055 Portici, Italy; gianluca.melone1998@gmail.com (G.M.); valentinomariaguastaferro@cnr.it (V.M.G.); giorgio.formisano@cnr.it (G.F.); massimo.giorgini@cnr.it (M.G.); 2 Department of Agricultural Sciences, University of Naples Federico II, 80055 Portici, Italy; lucia.andretta@unina.it (L.A.); eleonoraromito98@gmail.com (E.R.); 3 Center for Studies on Bioinspired Agro-Environmental Technology, BAT Center, University of Naples Federico II, 80055 Portici, Italy

**Keywords:** *Amitus spiniferus*, biological control, *Cales noacki*, citrus woolly whitefly, *COI* gene, *28S-D2* gene, DNA barcoding, *Eretmocerus paulistus*, integrated taxonomy, *Signiphora xanthographa*

## Abstract

**Simple Summary:**

The woolly whitefly *Aleurothrixus floccosus*, an invasive species in Italy, was first reported in 1974. Due to the limited efficacy of insecticides, its management relies primarily on classical biological control, notably through introducing the biological control agents *Cales noacki* and *Amitus spiniferus*. Approximately thirty years later, between 2024 and 2025, new surveys conducted in organic citrus orchards revealed a diversification of the parasitoid community associated with *A. floccosus*. These investigations confirmed the activity of two additional Neotropical species: *Eretmocerus paulistus* and *Signiphora xanthographa*. These findings represent new records for Italy and Europe, respectively. Their parasitic activity, distribution in various central and southern Italy locations, and ecological interactions with the host and among parasitoids are described.

**Abstract:**

The woolly whitefly, *Aleurothrixus floccosus*, is likely a Neotropical origin species that has spread globally. Introduced to France in 1969, it became a pest in southern European citrus groves, first reported in Italy in 1974. Integrated management using biological control agents is crucial due to the low efficacy of chemical controls. Nymphs produce waxy filaments and honeydew, limiting insecticide contact. Natural enemies, especially from Neotropics, have been documented. The parasitoids *Amitus spiniferus* and *Cales noacki* were released in France in 1970 and later observed in Liguria, Italy. In the Campania region, *C. noacki* was first found on *Aleurotuba jelineki* in 1984 and this finding preceded the first report of *A. floccosus* in the same area. Subsequently, *C. noacki* was also introduced in other regions where it showed better adaptation throughout the Italian territory, reaching high parasitization levels on the woolly whitefly nymphs. After many years since the last field investigations, surveys in 2024–2025 in organic citrus groves in central and southern Italy identified additional parasitoids. Besides *C. noacki* and *A. spiniferus*, *Eretmocerus paulistus* and *Signiphora xanthographa* were found for the first time in Italy. Both species were originally described from the Neotropical ecozone. The aphelinid finding represents its first documented establishment in Italy, while the signiphorid one represents a new record for the European fauna. *E. paulistus* is a primary parasitoid, while *S. xanthographa* is a hyperparasitoid that can limit the effectiveness of other parasitoids. The interaction of these parasitoids resulted in high parasitism rates for *A. floccosus* nymphs. Preserving the current complexity of parasitoids in integrated pest management (IPM) programs could effectively control the woolly whitefly in central and southern Italy.

## 1. Introduction

The woolly whitefly, *Aleurothrixus floccosus* (Maskell) (Hemiptera: Aleyrodidae), is a worldwide species, probably native to the Neotropics, where it is widely distributed. It is also present in Africa, mainly in the tropical zone, while in Asia, its presence has been recorded since 1994, and its distribution is currently restricted to a limited number of countries [1]. In Europe, *A. floccosus* was first reported in France in 1966 [2,3], spreading in the following years to Italy and other Mediterranean countries [1,4,5]. The woolly whitefly is a polyphagous species, with its host plants belonging to 31 botanical families, and a marked preference for citrus hosts [1,5]. Adult whiteflies prefer to feed and lay eggs on the underside of young leaves that emerge during active growth [6,7]. Damage is caused by the sap-sucking behavior of the nymphs, which also secrete honeydew on which sooty molds are stratified, extensively covering the leaves, causing a reduction in photosynthesis and gradually weakening the plants [8,9]. In Mediterranean countries the pest can develop 4–7 overlapping generations per year under favorable climatic conditions, causing economic damage to citrus production if control measures are not adopted [10,11,12]. Chemical control of *A. floccosus* is often ineffective due to the abundant wool-like wax filaments covering the nymphs, which hinder the penetration of contact insecticides [13]. An integrated management strategy that relies on the activity of natural enemies is essential to enhance the efficacy of control measures.

Several natural enemies of *A. floccosus* have been reported in the literature, including parasitoids, generalist predators and entomopathogenic fungi [14,15,16]. Among its numerous parasitoids, most species belong to the genera *Encarsia* Förster (Hymenoptera: Aphelinidae), *Cales* Howard (Hymenoptera: Calesidae), *Eretmocerus* Haldeman (Hymenoptera: Aphelinidae), and *Signiphora* Ashmead (Hymenoptera: Signiphoridae) [17]. Some species of the genus *Amitus* Haldeman (Hymenoptera: Platygastridae) are also reported [5]. The most efficient parasitoids of *A. floccosus* are generally considered to be *Cales noacki* Howard and *Amitus spiniferus* (Brèthes), which have been widely used in classical biocontrol programs [8,10,13,15,16,18,19,20,21,22,23,24,25]. The introduction of *C. noacki* reduced, under optimal environmental conditions, woolly whitefly populations by more than 95% from their original level [8,24,26]. In France, *C. noacki* and *A. spiniferus* were introduced as classical biological control agents in 1969 [27]. In Italy, both parasitoids were first recorded in the 1980s in Liguria, a northern region bordering France, from where they likely arrived, following the route of their host [18]. At the same time, in the Campania region (southern Italy), *C. noacki* was found on *Aleurotuba jelineki* Takahashi (Hemiptera: Aleyrodidae), which acted as a substitute host before the presence of *A. floccosus* was detected in that region [28]. Subsequently, *C. noacki* and *A. spiniferus* were introduced in Sicily [19]. Throughout Italy, *C. noacki* has demonstrated excellent adaptability, including on the islands, consistently achieving high parasitization rates on woolly whitefly nymphs [12,18,19,20,21,29,30,31]. As a key biological control agent against the woolly whitefly, *C. noacki* is extensively utilized in citrus-producing areas throughout Africa, the Mediterranean basin, and North America [32]. Field observations have revealed that the host range of *C. noacki* extends beyond the order Hemiptera [33]. In regions where *C. noacki* has been introduced, such as California, it appears to have displaced native parasitoid species that specifically attack *A. floccosus*, although this has not compromised the effectiveness of biological control efforts [22]. In contrast, in other areas of introduction, the displacement of various native parasitoids, including those not directly associated with *A. floccosus*, has also been hypothesized as a consequence of the establishment of *C. noacki* [34]. Concerns regarding its potential negative impact on native parasitoid communities led to its removal from the list of biological control agents, as recommended by the European and Mediterranean Plant Protection Organization (EPPO), in 2008 [35].

Since its initial detection in Italy in 1974, *A. floccosus* has caused significant damage to citrus crops across multiple regions [12]. Chemical control remains challenging, highlighting the need to monitor the presence and effectiveness of native natural enemies prior to implementing any management strategy. However, since the 1990s, limited research has been conducted on the dynamics of the parasitoid complex associated with *A. floccosus* in Italy and across Europe. The present study seeks to address this knowledge gap.

Over the past two years, more than three decades after the last studies on the biocenosis of *A. floccosus* in Italy, we conducted a field survey in two regions of southern and central Italy, respectively. In the citrus groves studied, we found a greater-than-expected diversity of parasitoid species attacking *A. floccosus*, including *C. noacki*, *A. spiniferus*, and two other species not previously reported in Italy. An integrated approach, which included morphological analysis and DNA barcoding, allowed us to ascribe them to *Eretmocerus paulistus* Hempel and *Signiphora xanthographa* Blanchard, respectively. We found a strong interaction among the four parasitoid species, which determines the high parasitization rate of *A. floccosus*. The impact of the parasitoid complex on the biological control of *A. floccosus* is discussed.

## 2. Materials and Methods

### 2.1. Field Collections

This study was carried out between 2024 and 2025 as part of the Phytosanitary Surveillance Project of the Campania Region, southern Italy, which monitored hundreds of sites to record, through occasional sampling, the presence and abundance of *A. floccosus* and its natural enemies on citrus plants in various contexts (specialized citrus orchards, private gardens, and public parks). The interaction between *A. floccosus* and parasitoid species was studied at two sites chosen for the absence of insecticide use. An intensive sampling plan was implemented in these two sites. Specifically, in Portici (Province of Naples), bimonthly sampling was conducted from March 2024 to February 2025 in an orchard of approximately one hundred lemon and clementine trees located within the Gussone Park, part of the Department of Agricultural Sciences at the University of Naples Federico II. This site was selected due to its accessibility and the total absence of phytosanitary treatments in the entire park. The second site was Casagiove (Province of Caserta), where sampling was carried out bimonthly from June to October 2024 in a family-run organic garden with twenty clementine trees. Further sampling was conducted in central Italy. The sole site, Grottammare (Province of Ascoli Piceno, Marche Region), was sampled occasionally between August and September 2024 on approximately thirty citrus plants used as urban trees, all of which were managed without any phytosanitary treatments. Information on the three sampled sites is reported in Table 1. In each site, at each sampling event, 20 fully developed leaves, infested by *A. floccosus*, were collected from newly developed shoots, on a variable number of trees depending on site-specific infestation levels. Each sample of leaves was sealed in double plastic bags, promptly transported to the laboratory, and stored under ventilated conditions until analysis.

### 2.2. Parasitization Rate and Phenology

Within 24–48 h of collection, the 20 infested leaves of each sample were examined under a stereomicroscope (Leica MZ16, Wetzlar, Germany). Nymphs of *A. floccosus* from the second to the fourth instar were counted, distinguishing between healthy nymphs and those showing signs of parasitization. Apparent parasitization was determined by the presence, visible by transparency, of preimaginal stages (larvae, pupae), or adults of primary parasitoids inside the host. Additional indicators included the abnormal localization of bacteriome, melanization of the oviposition hole, and, on the ventral side of the nymphs, the presence of parasitoid eggs or first-instar larvae, or the entry scar of ecto-endoparasitoids. Signs of hyperparasitism were recorded based on the observation of hyperparasitoid larvae or adults co-occurring with remains of the primary parasitoid within the host. The exit holes of adult parasitoids were not considered as a criterion for estimating parasitization, to avoid overestimating the active parasitization rates due to the possible overlap of different generations of the whitefly on the same leaf from which the apparently parasitized forms were isolated.

Parasitized nymphs were carefully detached from the leaves and stored individually in natural gelatin capsules (13.59 mm × 5.57 mm) under controlled laboratory conditions (25 ± 2 °C, 65 ± 10% RH, 16:8 L:D photoperiod). The primary host (*A. floccosus*) was confirmed by direct observation of the immature stages on the leaves and by the morphology of the emerged parasitoids. Parasitized nymphs were examined every 2–3 days, for about a month, for the emergence of adult parasitoids and hyperparasitoids. The timing and abundance of parasitoid emergence were recorded for each sampling event to assess the phenology of the study species.

The active parasitization rate, expressed as a percentage, was calculated for each sample of 20 leaves, according to Viggiani [36], using the formula:

$$\left[\frac{n{}^{\circ}\;\mathrm{apparently}\;\mathrm{parasitized}\;\mathrm{nymphs}}{n{}^{\circ}\;2^{th}\text{–}4^{th}\;\mathrm{instar}\;\mathrm{nymphs}}\right]\times 100$$

Dead nymphs and empty 4th instar nymphs from which the adult whitefly or adult parasitoid had already emerged were excluded from the count.

### 2.3. Morphological Analysis

Adult parasitoid specimens used for taxonomic analysis were slide-mounted in Canada balsam–phenol medium [37]. The specimens were examined and photographed, when necessary, under a microscope (Leica DMLS, Wetzlar, Germany). Dichotomous keys [33,38,39,40,41,42] were used to identify parasitoid species. They were also compared with the specimens deposited at the Filippo Silvestri Museum of the Department of Agricultural Sciences, University of Naples Federico II, Portici, Italy.

### 2.4. Molecular Analysis

Sample preparation and direct PCR amplification were performed following the protocol of Thongjued et al. [43], with minor modifications. Ethanol-preserved specimens were briefly rinsed in sterile distilled water, gently mixed, and air-dried on sterile filter paper. The entire body of each specimen was then transferred individually into a PCR tube containing 10 µL of 1× phosphate-buffered saline (PBS) and incubated at 98 °C for 3 min in a MiniAmp Thermal Cycler (Applied Biosystems™) to facilitate DNA release. Furthermore, DNA was also extracted from individual insects using a conservative Chelex-proteinase K-based protocol as in Gebiola et al. [44] to have specimens for subsequent morphological analysis. The mitochondrial cytochrome c oxidase subunit I (*COI*) gene region was amplified using the primers LepF1/LepR1 [45] (~700 bp), targeting the barcoding *COI* region, or C1-J-2183/TL2-N-3014 [46], targeting the 3′ terminal region (~800 bp), with thermocycling conditions as described in Warbroeck et al. [47]. The 28S-D2 rRNA gene region (~650 bp) was amplified using the primers D2F/D2R [48], following PCR conditions described by Guerrieri et al. [49]. PCR products were purified using the Monarch^®^ PCR & DNA Cleanup Kit (New England Biolabs, Ipswich, MA, USA) and sequenced by Eurofins Genomics Europe Shared Services GmbH (Ebersberg, Germany). Bidirectional sequencing chromatograms were edited and aligned using ChromasPro 1.7.6 (Technelysium Pty Ltd., South Brisbane, QLD, Australia) and AliView 1.28 software (Uppsala, Sweden) [50], respectively. Final consensus sequences were queried against the GenBank (www.ncbi.nlm.nih.gov/genbank/; accessed on 25 August 2025) and Barcode of Life Data System (BOLD; www.boldsystems.org; accessed on 25 August 2025) databases.

## 3. Results

### 3.1. Morphological Analysis

**Figure 1:**
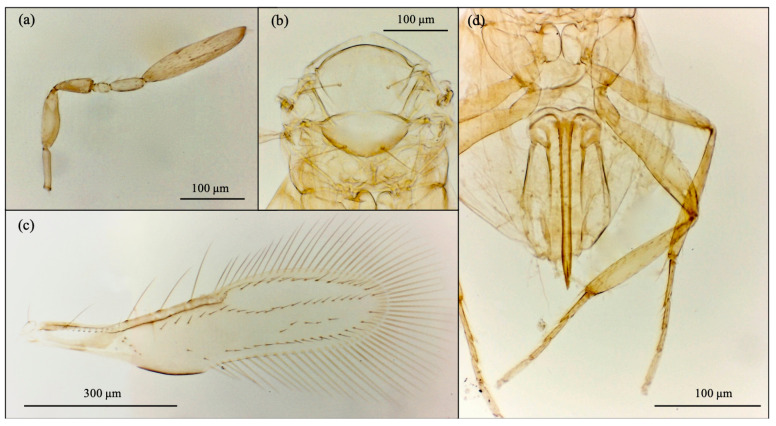
*Cales noacki*: female. (**a**) antenna; (**b**) thorax; (**c**) forewing; (**d**) ovipositor, middle and hind legs.

**Figure 2:**
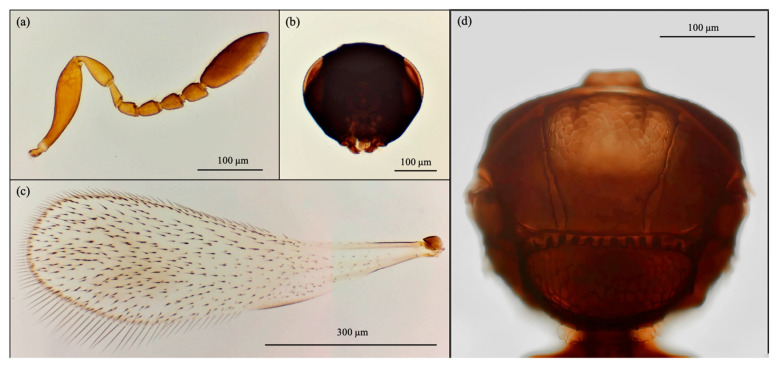
*Amitus spiniferus*: female. (**a**) antenna; (**b**) head; (**c**) forewing; (**d**) thorax.

**Figure 3:**
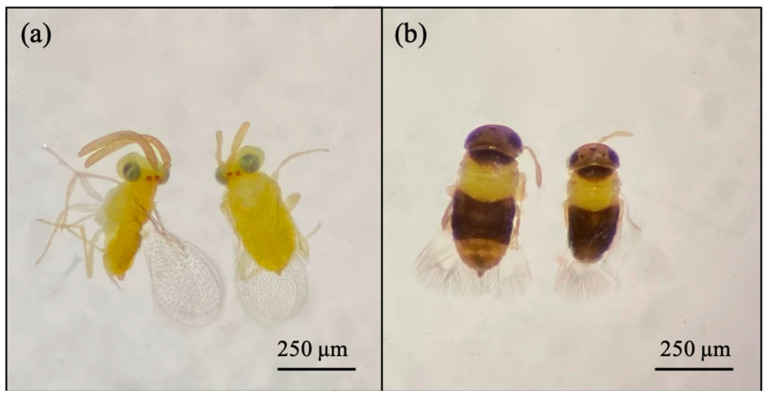
(**a**) *Eretmocerus paulistus* female (right) and male (left); (**b**) *Signiphora xanthographa* female (left) and male (right).

**Figure 4:**
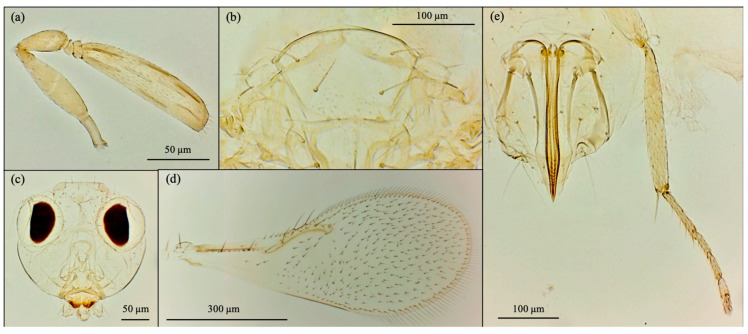
*Eretmocerus paulistus* female. (**a**) antenna; (**b**) mesoscutum; (**c**) head; (**d**) forewing; (**e**) ovipositor, midtibia and midtarsi.

**Figure 5:**
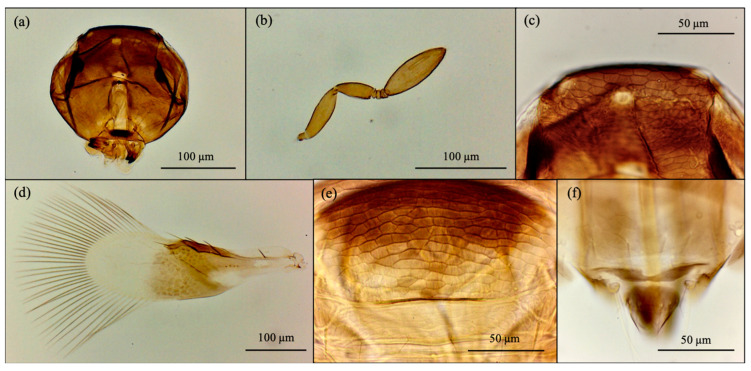
*Signiphora xanthographa* female. (**a**) head; (**b**) antenna; (**c**) vertex sculpture; (**d**) forewing; (**e**) mesoscutum sculpture; (**f**) medial incision on the eighth urotergite.

Based on morphological features, the emerged parasitoid species were identified as *Cales noacki* (Hymenoptera: Calesidae) (Figure 1), *Amitus spiniferus* (Hymenoptera: Platygastridae) (Figure 2), *Eretmocerus paulistus* (Hymenoptera: Aphelinidae) (Figure 3a and Figure 4) and *Signiphora xanthographa* (Hymenoptera: Signiphoridae) (Figure 3b and Figure 5). At the Casagiove site, a notable prevalence of the last two species was observed, and only one female of *C. noacki* was recovered. In Portici (NA), the four species were simultaneously present, while in Grottammare (AP), only the presence of the signiphorid was not recorded.

*E. paulistus* (Figure 3 and Figure 4) and *S. xanthographa* (Figure 3 and Figure 5) represent new records for the Italian and European fauna, respectively.

According to Rose [39], the female of *E. paulistus* collected in Italy (Figure 4) is characterized by the antennal club being relatively short, approximately 3.6 times as long as it is wide and broadly spatulate in shape (Figure 4a). Additional diagnostic features are the second funicle article compressed (Figure 4a) and the midlobe of the mesoscutum bearing four setae (Figure 4b). This species closely resembles *E. debachi* (Rose & Rosen), E. *furuhashi* (Rose & Zolnerowich), and *E. desantisi* Rose [39]; however, *E. paulistus* can be distinguished from these species by the morphology of the antennal club.

According to Woolley and Dal Molin [42], the females of *S. xanthographa* collected in Italy (Figure 5) are characterized by the short antennal club and the body uniformly light brown (Figure 5b). The head is slightly paler on the frons and shows strong reticulation on the vertex (Figure 5a,c). The mesoscutum is brown centrally and anteriorly, yellow to pale yellow posteriorly and laterally, with reticulated sculpturing on the anterior region and midlobe (Figure 5e). The scutellum and metanotum are pale yellow. Metasoma coloration is taxonomically significant and varies among different urotergites, from light brown to dark brown or yellow. However, it presents intraspecific variability: some individuals have a completely yellow or uniformly brown metasoma. Furthermore, the eighth urotergite of the metasoma features a rounded median incision (Figure 5f). The forewing is infuscate to just beyond the stigmal vein, with hyaline areas at the base; the marginal vein bears a seta (Figure 5d). Males are easily recognized by their entirely brown metasoma (Figure 5b). *Signiphora xanthographa* resembles *S. coquilletti* Ashmead, *S. aleyrodis* Ashmead, and *S. flavella* Girault, but differs in antennal club shape, vertex and mesoscutum sculpturing, and male coloration. Both *S. coquilletti* and *S. aleyrodis* exhibit transverse striations on the vertex and frons, with males showing pale yellow coloration on the fifth and sixth urotergites. *Signiphora flavella* lacks the medial incision on the eighth urotergite of the metasoma, which is transversely margined [42].

### 3.2. Molecular Analysis

We did not find sequences of *E. paulistus*, *S. xanthographa* and *A. spiniferus* deposited in the public databases GenBank and BOLD. Following sequence trimming, *COI* sequences of 792 bp of the 3′ terminal region, and of 652 bp and 649 bp of the barcoding region were obtained for *E. paulistus*, *S. xanthographa*, and *A. spiniferus,* respectively.

The BLAST 2.12.0 analysis of the *E. paulistus COI* sequence revealed the highest identity (96.72%) with *E. desantisi* (GenBank accession: EU017332.1). In the BOLD database, the closest match was with *Eretmocerus* sp. (Sequence ID: GBMIN71576-17), showing 91.50% similarity. The *28S-D2* sequence of *E. paulistus* showed the highest identity (98.67%) with an *Eretmocerus* sp. (GenBank accession: JN623551.1), followed by *E. eremicus* Rose & Zolnerowich (96.52%, accession number AY599369).

The *COI* sequence of *S. xanthographa* showed 100% identity with *Signiphora* sp. in both GenBank (accession number MH456749.1) and BOLD (Sequence ID: GBMNA20244-19); other closest *Signiphora* species (*S. flavella* Girault MH456540, *S. perpauca* Girault MH456745 and *S. bifasciata* Ashmead MH456703) showed 87.7% identity. The *28S-D2* sequence of *S. xanthographa* showed the highest identity (97.72%) with *S. aleyrodis* Ashmead (GenBank accession: AY599343.1).

The *COI* sequence of *A. spiniferus* showed 89.64% identity with *A. bennetti* Viggiani & Evans (GenBank accession: OM387006.1), while BOLD searching returned a match only at the family level (Platygastridae). The *A. spiniferus 28S-D2* sequence showed the highest identity (94.07%) with *A. hesperidum* Silvestri (GenBank accession: PQ461856.1).

The *28S* rDNA sequence of *C. noacki* showed 100% identity with a reference *C. noacki* sequence (GenBank accession: GQ374777.1).

Sequences of the parasitoid species studied were deposited in GenBank (Table 2) and the respective voucher specimens (slide-mounted preparations) were deposited at the Museum of Entomology “F. Silvestri”, University of Naples “Federico II”, Portici, Italy.

### 3.3. Parasitization Rate

**Figure 6:**
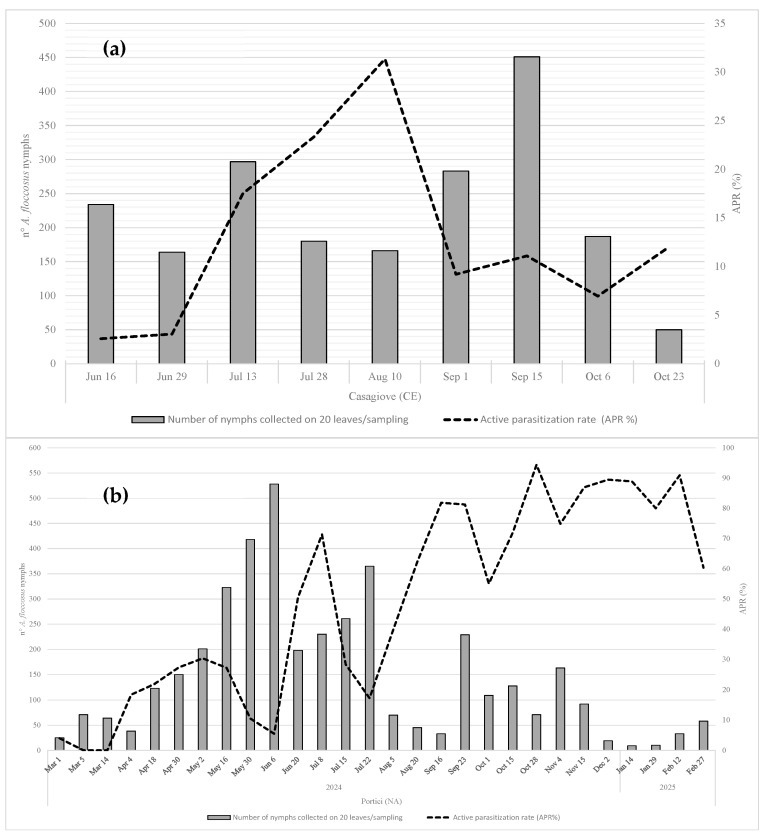
Population dynamics of *A. floccosus* and the active parasitization rate during 2024–2025: (**a**) Casagiove site; (**b**) Portici site.

At the Casagiove site (Figure 6a), the active parasitization rate (APR%) oscillated between 2.56% and 3.05% during the month of June. Subsequently, there was a gradual increase until reaching a peak (31.33%) on the 10th of August. A reduction in the APR was recorded in the second half of August, and at the beginning of September, it stood at 9.19% before rising slightly in October.

At the Portici site (Figure 6b), after an initial low level of parasitoid activity recorded in March, with an APR that fluctuated between 4% and 0%, an increase in parasitoid activity was observed in April, culminating in a peak (30.35%) recorded on the 2nd of May. Subsequently, a new reduction in the APR, which stands at 5.49%, was observed on the 6th of June. A new rise in the active parasitization rate was observed from late June to early July, reaching a notable peak of 71.30%. This was followed by a sharp decline on July 22nd, with an APR of 17.26%. Subsequently, parasitoid activity increased steadily, with significant peaks ranging from 81% to 91% between mid-September and February. In Grottammare, the APR recorded in the two occasional samplings was 28.67% on the 20th of August and 40.59% on the 30th of September.

Additional information about the incidence of each parasitoid on the parasitization levels is available in Supplementary Material (Tables S1 and S2).

### 3.4. Phenology

**Figure 7:**
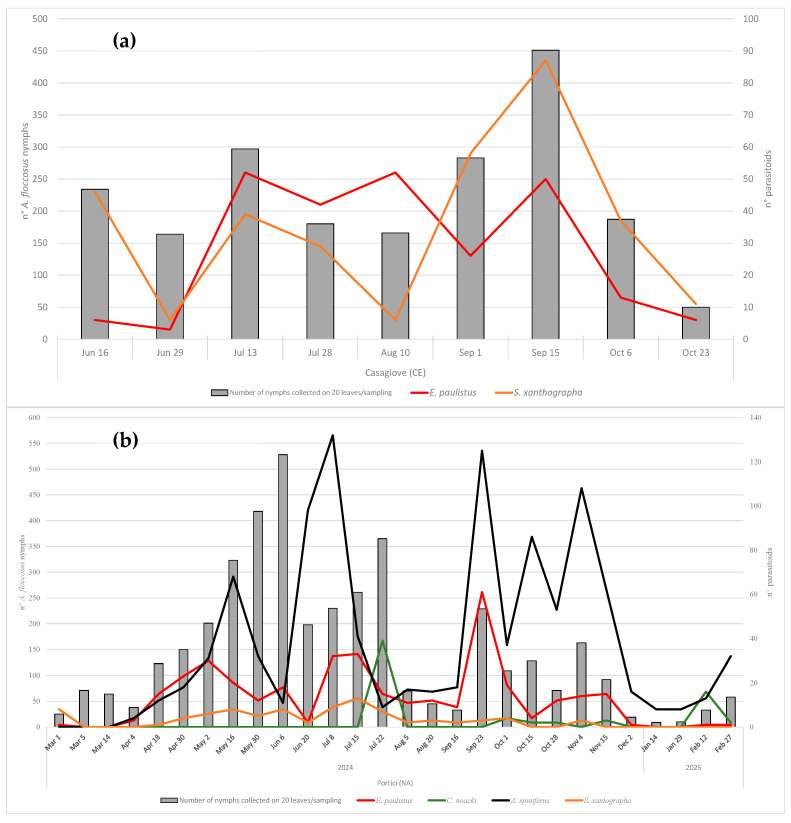
Population dynamics of *A. floccosus* and its parasitoids during 2024–2025: (**a**) Casagiove site; (**b**) Portici site.

The population dynamics of *A. floccosus* and its parasitoid species are shown in Figure 7. In Casagiove (CE), data show oscillating trends in the months of June and July, with a peak of presence reached on the 13th of July. *Eretmocerus paulistus* and *S. xanthographa* are the only active species in the monitoring area, except for a single female of *C. noacki.* Between the end of July and August, the populations of the woolly whitefly and *S. xanthographa* decreased and reached the lowest values observed during the summer, while the *E. paulistus* populations remained numerically constant. In the following phase, from the early part of September to the middle of the same month, the woolly whitefly population increased and reached a new maximum on the 15th of September with 451 nymphs counted. During the same period, the presence of both parasitoids increased, but *S. xanthographa* developed a larger population than *E. paulistus*. Since October, the woolly whitefly population has decreased, and overall, a minimum value of 50 nymphs was observed. Similarly, a reduction in the parasitoid number was observed for *S. xanthographa*, maintaining higher values than *E. paulistus,* as in the previously observed period.

In Portici (NA), the population of *A. floccosus* gradually increased during the spring period until the beginning of June, when the infestation of the woolly whitefly reached an initial peak with 528 nymphs counted. During the same period, the populations of parasitoids gradually increased until the 16th of May, followed by a reduction during late spring and the beginning of summer. In this phase, *A. spiniferus* and *E. paulistus* prevailed over *S. xanthographa*, while *C. noacki* was never detected. Subsequently, after an initial decrease, the infestation of *A. floccosus* gradually increased in July and a considerable prevalence of *A. spiniferus* was observed compared to the other parasitoids. At the same time, there was an increase in *E. paulistus* and *S. xanthographa* populations, but with a greater incidence of the former. The presence of *C. noacki* became evident only in the second half of July, reaching its peak on July 22nd with 39 individuals collected. During this period, *C. noacki* was more abundant than any other species observed. A considerable reduction for all populations, host and parasitoids, occurred in midsummer, followed by a new increase in September. During this period, *A. spiniferus* again reached a population peak, with 229 nymphs counted. In the same period, *E. paulistus* reached its maximum peak for the entire monitoring period, equal to 61 individuals collected. In autumn, the whitefly infestation followed a sinusoidal trend, with only a few nymphs observed, peaking on November 4th with 163 individuals recorded. During this phase, *A. spiniferus* was the most abundant parasitoid, followed by *E. paulistus*. In contrast, no increase was observed in the populations of *S. xanthographa* and *C. noacki*. In late autumn and the early winter months, the lowest levels of *A. floccosus* infestation were recorded, with an average of 37 individuals. During this period, the presence of its natural enemies was also minimal, averaging only 8.7 individuals.

In Grottammare 422 and 303 nymphs of *A. floccosus* were counted on the 20th of August and 30th of September, respectively. At this site, *C. noacki* was the dominant parasitoid, with an average of 62.5 individuals, followed by *A. spiniferus* with 38.5 individuals. At the same site, the signiphorid was not detected, while the presence of *E. paulistus* was confirmed, averaging 21 individuals.

Additional information about the abundance of each parasitoid is available in the Supplementary Materials (Tables S1 and S2).

## 4. Discussion

During 2024 and 2025, field observations confirmed the presence of *C. noacki* in all the sites investigated. The integration of morphological and molecular analyses for the identification of parasitic Hymenoptera provides a robust and reliable approach to improving taxonomic accuracy [51,52]. Despite persistent uncertainty and instability surrounding the classification of the genus *Cales*, particularly its type species *C. noacki*, keys were proposed for the identification of both male and female specimens within the *C. noacki* group [33]. However, some individuals of both sexes may exhibit cryptic morphological traits that are indistinguishable from those of at least one other species. Given these limitations, the authors emphasized the importance of molecular tools for achieving reliable species-level identification. Consequently, the specimens we collected in central and southern Italy were assigned to the *C. noacki* group, and molecular analysis confirmed the morphological identification of *C. noacki*. For *A. spiniferus*, although we did not find reference sequences in public databases, the taxonomy based on morphological characters remains well-defined and unequivocal.

The aphelinid *E. paulistus* was originally described by Hempel in 1904 [53] based on one female and two male specimens reared from *A. floccosus* collected on citrus plants in Campinas, Brazil. Its known geographical distribution includes Argentina, Brazil, California, Chile, Cuba, Haiti, Iran, Mexico, Peru, Spain, and India [54]. In this study, we report the presence of *E. paulistus* in Italy for the first time. *Eretmocerus paulistus* is a primary parasitoid of whitefly species and its known hosts include *A. floccosus*, *Aleyrodes horridus* Hempel and *Neomaskellia bergii* (Signoret) [54]. GenBank and BOLD public databases did not return any reference sequences of *E. paulistus*. Its *COI* sequence shared 96.72% identity with *E. desantisi*, which is morphologically like *E. paulistus*. Therefore, DNA barcoding supports the distinction between the two species based on morphological characters.

*Signiphora xanthographa* was originally described by Blanchard in 1936 [55] based on two female and three male specimens reared from *A. floccosus* collected in Argentina. Its known distribution includes Argentina, Brazil, Chile, China, Colombia, Peru, Thailand, Trinidad and Tobago, and Uruguay [42,54]; in this study, we report the presence of *S. xanthographa* in Europe for the first time. This species exhibits hyperparasitic behavior, developing on the primary parasite of whiteflies [42]. However, most records of *S. xanthographa* refer to the primary host, including mainly whiteflies (*Aleurotrixus* sp., *Aleyrodes* sp. and *Tetraleurodes* sp.) and an armored scale insect, and not only to the parasitoid host [42,56,57]. It has been shown that *S. xanthographa* is a hyperparasitoid of *A. spiniferus* in *A. floccosus* and is able to oviposit only when females are provided with parasitized nymphs [42]. Based on our observations, *S. xanthographa* appears to act predominantly as a hyperparasitoid. The *COI* sequence obtained from *S. xanthographa* specimens collected in southern Italy exhibited 100% identity with an unidentified *Signiphora* species, indicating a likely close taxonomic relationship. However, unlike *S. xanthographa*, *Signiphora* sp. was recorded as a hyperparasitoid not of whiteflies but of the scale insect *Lepidosaphes beckii* (Newman) on *Citrus limon* in Chile [58]. *Signiphora xanthographa* was also recorded as emerging by the armored scale *Chrysomphalus aonidum* (L.) [42]. Whether *S. xanthographa* collected in Italy, which acts as a hyperparasitoid on *A. floccosus*, could also behave as a hyperparasitoid or primary parasitoid of Diaspididae species is a hypothesis that needs confirmation.

The collected and identified parasitoids warrant further consideration from an ecological and biocontrol perspective. The use of *Eretmocerus* species in biological control programs targeting the woolly whitefly has been well documented, beginning with the introduction of *E. paulistus* from Mexico to the United States [8,22]. This species is the dominant parasitoid of *A. floccosus* in several regions of Mexico and has also demonstrated effective natural control in other South American countries [8,59,60,61]. An updated identification key was provided for *Eretmocerus* species collected from *A. floccosus* during surveys conducted in Central and South America [39]. Among the listed species, only *E. paulistus* has been introduced into Spain, alongside *C. noacki* and *A. spiniferus* [16,39,62,63]. However, *E. paulistus* failed to establish in this country [64,65].

The literature provides limited insights into the phenology and parasitic trophic interactions of *Signiphora* species associated with *A. floccosus.* In most cases, unidentified *Signiphora* species have been reported as parasitoids or hyperparasitoids of the woolly whitefly in South America [53,60,61,66,67,68,69,70]. In Europe, *S. townsendi* Ashmead, later synonymized with *S. aleyrodis* [42], has been reported as a probable hyperparasite of *C. noacki* and *A. spiniferus*, both associated with the woolly whitefly in Sicily [71]. Moreover, *S. aleyrodis* has been cited in the literature as a hyperparasitoid of *Eretmocerus* sp. associated with *A. floccosus* [69,70]. More recently, *S. flavella* was recorded in Greece as a parasitoid of the same host, with populations surpassing those of *C. noacki* [72], although its trophic role remains unclear. Our observations indicate that *S. xanthographa* exhibits hyperparasitism activity against all primary parasitoids, particularly when their populations are abundant. The regulatory effect of the hyperparasitoid on the population size of primary parasitoids is particularly evident where *E. paulistus* is the only parasitoid, whereas it appears less evident against *A. spiniferus*.

Very little is known in the literature about the biological characteristics of *A. spiniferus*, particularly its impact on pest populations in areas where it has been introduced for classical biological control. In our study, its control activity is remarkably evident at least in one site, Portici, where it represents the most abundant parasitoid species. 

The presence of *C. noacki* was also noted, although not uniformly in all the areas sampled and always at low population levels compared to the other parasitoid species. The latter, contrary to what is reported in the literature [22], do not appear to be limited by the presence of *C. noacki*.

In both Portici and Casagiove, the population dynamics of the woolly whitefly followed similar trends. Infestation levels increased during spring and early summer, likely in response to warmer temperatures and the emergence of young foliage, accompanied by a rise in parasitization rates. A marked decline in whitefly populations was observed in midsummer, followed by a resurgence in September. During autumn, both whitefly and parasitoid populations fluctuated before reaching minimum levels in winter. According to Onillon and Abbassi [10], cold temperatures slow development and are likely to cause direct mortality of immature whiteflies.

The effectiveness of parasitoid-mediated pest control was particularly evident in Casagiove, where the highest parasitization rate was recorded in August, coinciding with the lowest observed host density. In contrast, parasitization rates in Casagiove during the autumn fluctuated between 11% and 12%. This difference is likely due to the higher incidence of hyperparasitism by *S. xanthographa* on *E. paulistus*. Similar patterns have been reported in Chile, where the simultaneous presence of *C. noacki*, *A. spiniferus*, *E. paulistus*, and a larger population of *Signiphora* spp. resulted in an average parasitization rate of 15% [61]. In Grottammare, observations confirm the presence of *E. paulistus* in the Marche region. The APR on August 20th (28.67%) was comparable to that of Casagiove during the same period, while the rate on the 30th of September (40.59%) was more in line with that recorded in Portici on the 1st of October. The higher parasitization rate observed in Grottammare in September, compared to Casagiove, is likely due to the absence of *S. xanthographa*, which was not detected at this site.

Given the frequent association of *E. paulistus* and *S. xanthographa* with *A. floccosus* in Central and South America [39,42,55], the polyphagous nature of *A. floccosus*, and the fact that commercial citrus imports typically involve defoliated fruits, it is plausible that both natural enemies were accidentally introduced through the trade of ornamental plants carrying parasitized hosts. Global trade and international travel are widely recognized as major pathways for introducing non-native organisms into new ecosystems [73,74].

The data obtained in this survey suggest that, within the surveyed area and in the presence of exclusively exotic parasitoids, *C. noacki* does not exert a negative impact on the local biocoenosis, nor does it compromise the effectiveness of biological control against invasive populations of *A. floccosus*. On the contrary, the current parasitoid community associated with the citrus woolly whitefly, if preserved and integrated into pest management programs, may continue to provide effective control of the pest in central and southern Italy. Under present conditions, any claims regarding the potential negative impact of *C. noacki* on native entomophagous insects, which led to its removal from the EPPO Positive List, remain speculative and lack empirical support. Like *C. noacki* and *A. spiniferus*, *E. paulistus* is a primary parasitoid of the woolly whitefly, although it exhibits ecto-endophagous trophic behavior. Observations conducted on sampled material, consistent with [42], also confirmed that *S. xanthographa* acts as a hyperparasitoid and, in the examined areas, can develop on all parasitoid species associated with *A. floccosus*. Although our observations did not yield concrete evidence that *E. paulistus* and *S. xanthographa* compromise the effectiveness of endophagous primary parasitoids in pest control, future studies will assess the impact of different trophic behaviors. Additional research will investigate the distribution of parasitoids associated with *A. floccosus*, aiming to understand the influence of altitudinal, geographical, and climatic factors.

## Figures and Tables

**Table 1:** Sampling localities in Southern and Central Italy.

Localities	Coordinates	Host Plants
Casagiove (CE)	41.078472 N, 14.316778 E	Clementine *Citrus clementina* Hort. ex Tanaka
Portici (NA)	40.810556 N, 14.341944 E	Lemon *Citrus limon* (L.) Burm. f. Clementine *Citrus clementina* Hort. ex Tanaka
Grottammare (AP)	42.988778 N, 13.869278 E	Bitter orange *Citrus × aurantium* L. Orange *Citrus sinensis* (L.) Osbeck

**Table 2:** Specimen details, including sampling date, location, and associated sequence GenBank accession numbers.

Parasitoid Species	Localities	Coordinates	Date of Sampling	*COI* Accession Number	*28S-D2* Accession Number
*Eretmocerus paulistus*	Casagiove (CE)	41.078472 N, 14.316778 E	01.IX.2024	PX103238	PX103257
*Signiphora xanthographa*	Casagiove (CE)	41.078472 N, 14.316778 E	01.IX.2024	PX103239	PX103258
*Amitus spiniferus*	Portici (NA)	40.810556 N, 14.341944 E	22.VII.2024	PX118932	PX121923
*Cales* *noacki*	Portici (NA)	40.810556 N, 14.341944 E	22.VII.2024		PX121924

## Data Availability

All relevant data are within the paper.

## Supplementary Materials

The following supporting information can be downloaded at: https://www.mdpi.com/article/10.3390/insects16101037/s1, Table S1: Infestation, number of parasitoids and parasitization data (APR %) in the locality of Portici (NA); Table S2: Infestation, number of parasitoids and parasitization data (APR %) in the localities of Casagiove (CE) and Grottammare (AP); Figure S1. Infestation, number of parasitoids and parasitization data (APR %) in the locality of Portici (NA).
