# Supplementary material for: The Parasitoid Complex of Aleurothrixus floccosus (Hemiptera: Aleyrodidae) in the Citrus Groves of Central–Southern Italy
Source: Insects. 2025 Oct 9;16(10):1037. doi: 10.3390/insects16101037 (PMC12564006; doi:10.3390/insects16101037)
Supplement: Supplementary file 1 [file insects-16-01037-s001.zip › insects-3874411-supplementary.pdf]

Luogo		Data	*n. A. floccosus nymphs*	*E. paulistus*	*C. noacki*	*A. spiniferus*	*S. xantographa*	Active parasitization rate (APR)	Active parasitization rate (APR) by E. paulistus	Active parasitization rate (APR) by C. noacki	Active parasitization rate (APR) by A. spiniferus
Portici (NA)	2024	Mar 1	25	1	0	0	8	4.00	4.00	0.00	0.00
		Mar 5	71	0	0	0	0	0.00	0.00	0.00	0.00
		Mar 14	64	0	0	0	0	0.00	0.00	0.00	0.00
		Apr 4	38	3	0	4	0	18.42	7.89	0.00	10.53
		Apr 18	123	15	0	12	1	21.95	12.20	0.00	9.76
		Apr 30	150	23	0	18	4	27.33	15.33	0.00	12.00
		May 2	201	30	0	31	6	30.35	14.93	0.00	15.42
		May 16	323	20	0	68	8	27.24	6.19	0.00	21.05
		May 30	418	12	0	32	5	10.53	2.87	0.00	7.66
		Jun 6	528	18	0	11	8	5.49	3.41	0.00	2.08
		Jun 20	198	2	0	98	2	50.51	1.01	0.00	49.49
		Jul 8	230	32	0	132	9	71.30	13.91	0.00	57.39
		Jul 15	261	33	0	41	13	28.35	12.64	0.00	15.71
		Jul 22	365	15	39	9	7	17.26	4.11	10.68	2.47
		Aug 5	70	11	0	17	2	40.00	15.71	0.00	24.29
		Aug 20	45	12	0	16	3	62.22	26.67	0.00	35.56
		Sep 16	33	9	0	18	2	81.82	27.27	0.00	54.55
		Sep 23	229	61	0	125	3	81.22	26.64	0.00	54.59
		Oct 1	109	19	4	37	4	55.05	17.43	3.67	33.94
		Oct 15	128	4	2	86	0	71.88	3.13	1.56	67.19
		Oct 28	71	12	2	53	0	94.37	16.90	2.82	74.65
		Nov 4	163	14	0	108	3	74.85	8.59	0.00	66.26
		Nov 15	92	15	3	62	0	86.96	16.30	3.26	67.39
		Dec 2	19	1	0	16	0	89.47	5.26	0.00	84.21
	2025	Jan 14	9	0	0	8	0	88.89	0.00	0.00	88.89
		Jan 29	10	0	0	8	0	80.00	0.00	0.00	80.00
		Feb 12	33	1	16	13	0	90.91	3.03	48.48	39.39
		Feb 27	58	1	2	32	0	60.34	1.72	3.45	55.17

Table S1. Infestation, number of parasitoids and parasitization data (APR %) in the locality of Portici (NA)

Luogo		Data	*n. A. floccosus nymphs*	*E. paulistus*	*S. xanthographa*	*C. noacki*	*A. spiniferus*	Active parasitization rate (APR) by E. paulistus	Active parasitization rate (APR) by C. noacki
Casagiove (CE)	2024	Jun 16	234	6	46	0	0	2.56	0.00
		Jun 29	164	3	6	1	0	2.44	0.61
		Jul 13	297	52	39	0	0	17.51	0.00
		Jul 28	180	42	29	0	0	23.33	0.00
		Aug 10	166	52	6	0	0	31.33	0.00
		Sep 1	283	26	58	0	0	9.19	0.00
		Sep 15	451	50	87	0	0	11.09	0.00
		Oct 6	187	13	37	0	0	6.95	0.00
		Oct 23	50	6	11	0	0	12.00	0.00

Luogo		Data	*n. A. floccosus nymphs*	*E. paulistus*	*S. xanthographa*	*C. noacki*	*A. spiniferus*	Active parasitization rate (APR)	Active parasitization rate (APR) by E. paulistus	Active parasitization rate (APR) by C. noacki	Active parasitization rate (APR) by A. spiniferus
Grottammare (AP)	2024	20-ago	422	12	0	71	38	28.67	2.84	16.82	9.00
		30-set	303	30	0	54	39	40.59	9.90	17.82	12.87

Table S2. Infestation, number of parasitoids and parasitization data (APR %) in the localities of Casagiove (CE) and Grottammare (AP)
